# Prevalence of hypertension and cardiovascular risk in patients with gastrointestinal angiodysplasia: the role of aortic stiffness and non-dipper blood pressure patterns; predictive value of novel inflammatory biomarkers

**DOI:** 10.3389/fcvm.2025.1561853

**Published:** 2025-08-26

**Authors:** Abdulkadir Çakmak, Ömer Kertmen, Şirin Çetin

**Affiliations:** ^1^ Departmant of Cardiology, Faculty of Medicine, Amasya, Amasya, Türkiye; ^2^ Departmant of Biostatistics, Faculty of Medicine, Amasya, Amasya, Türkiye

**Keywords:** aortic stiffness, gastrointestinal angiodysplasia, left ventricular hypertrophy, nondipper hypertension, novel inflammatory markers

## Abstract

Gastrointestinal angiodysplasia (GIAD) is the most prevalent vascular malformation of the gastrointestinal (GI) tract, primarily affecting elderly individuals, with the colon being the most common site of involvement. Patients with GIAD often present with cardiovascular comorbidities such as hypertension, increased aortic stiffness, and left ventricular hypertrophy (LVH). This study aimed to evaluate the prevalence of hypertension and its association with aortic stiffness in patients diagnosed with GIAD with particular attention to the role of non-dipper blood pressure patterns. Additionally, the predictive value of inflammatory biomarkers—including the C-reactive protein to albumin ratio (CAR), blood urea nitrogen to albumin ratio (BAR), neutrophil-to-lymphocyte ratio (NLR), lymphocyte-to-C-reactive protein ratio (LCR), and systemic immune response index (SIRI) was investigated concerning vascular risk and disease pathophysiology. A total of 25 patients with GIAD and 25 matched controls were included. All participants underwent comprehensive cardiovascular evaluation, including physical examination, electrocardiography, transthoracic echocardiography, and 24 h ambulatory blood pressure monitoring (ABPM). Demographic characteristics, biochemical parameters, echocardiographic indices, and ABPM findings were analyzed. Receiver operating characteristic (ROC) curve analysis, area under curve (AUC) and multivariate regression models were used to assess the predictive value of inflammatory biomarkers. GIADs were predominantly localized in the colon (40%). Compared to controls, patients exhibited significantly increased aortic stiffness, greater aortic systolic and diastolic diameters, and a higher prevalence of LVH. The non-dipper blood pressure pattern was notably more frequent among patients (84% vs. 4%, *p* < 0.0001). Serum creatinine and potassium levels were significantly elevated in the patient group. Among inflammatory biomarkers, CAR (AUC: 0.70), BAR (AUC: 0.81), and NLR (AUC: 0.69) demonstrated the strongest associations with GIAD, with elevated CAR and BAR levels increasing disease risk by 4- to 6-fold. This study underscores the significant cardiovascular burden in patients with GIAD, characterized by hypertension, increased aortic stiffness, LVH, and a high prevalence of non-dipper blood pressure patterns. The integration of inflammatory biomarkers such as CAR and BAR may enhance early diagnosis and facilitate personalized management strategies. However, the limited sample size, single-center design and focus on only specific markers in our study may limit the generalizability of the results.

## Introduction

Gastrointestinal angiodysplasia (GIAD) represents a broad term encompassing the general concept of abnormal vascular formation and is recognized as one of the most frequently encountered vascular malformations within the gastrointestinal (GI) system (1–3). This condition is typically characterized by lesions consisting of dilated and tortuous capillaries, usually measuring less than 5 mm in diameter, predominantly localized within the mucosal and submucosal layers of the GI tract (2). These vascular anomalies predominantly occur in elderly populations, with a particular predilection for the cecum and ascending colon, and manifest along a wide clinical continuum, ranging from asymptomatic presentations to life-threatening GI hemorrhages (4, 5). Although GIADs can cause serious, life-threatening bleeding, it is not fully understood why some patients experience this bleeding while others do not (6). A study conducted in Korea found that the risk of bleeding was higher in angiodysplasia larger than 1 cm or located outside the antral region (gastric body/fundus), and upper gastrointestinal bleeding due to GIAD was more common in elderly patients (7). While endoscopic intervention remains the gold standard for both the diagnosis and management of angiodysplasia, other modalities such as computed tomographic (CT) angiography, radiolabeled red blood cell imaging, and surgical intervention may be required in certain cases (8).

In patients diagnosed with GIAD, the incidence of vascular comorbidities, including hypertension, ischemic heart disease, arrhythmias, valvular heart disease, heart failure, chronic kidney disease, and venous thromboembolism, is significantly elevated in comparison to the general population (9). The relationship between hypertension and aortic stiffness in patients with GIAD is a critical area of investigation, as both conditions significantly exacerbate vascular risk (10). Aortic stiffness is characterized by diminished arterial compliance, which leads to increased impedance to blood flow and elevated pulse wave velocity (11). While aortic stiffening is often considered a physiological consequence of aging, the coexistence of hypertension accelerates this process, compounding vascular compromise (12).

A critical aspect to consider in the context of GIAD is the prevalence of a non-dipper blood pressure pattern among affected patients. The non-dipper profile is defined by the absence of the typical nocturnal decline in blood pressure, a phenomenon commonly observed in healthy individuals (13). This deviation from the expected circadian rhythm of blood pressure regulation has been linked to an increased cardiovascular risk profile, which includes heightened aortic stiffness and a greater degree of target organ damage (14, 15). Patients with a non-dipper profile tend to have persistent vascular stress, which exacerbates endothelial dysfunction and contributes to the perpetuation of vascular problems. The prevalence of non-dipper hypertension in GIAD patients may thus represent an additional layer of cardiovascular complexity that needs to be addressed in their clinical management. Changes in inflammatory markers have recently been widely used to predict vascular diseases. Systemic immune response index (SIRI) is a good indicator of the balance between inflammatory capacity and *in vivo* inflammatory response. The SIRI is calculated by dividing the product of the absolute neutrophil count (10^3^/µl) and monocyte count (10^3^/µl) by the lymphocyte count (10^3^/µl). It is a strong predictor of vascular clinical processes such as stroke-associated pneumonia, coronary artery disease, and cerebrovascular events (16). Neutrophils intensify the inflammatory reaction by secreting various inflammatory mediators, while lymphocytes simultaneously suppress the inflammatory response. It has been shown that the neutrophil-to-lymphocyte ratio (NLR), calculated based on these two elements, is associated with the severity of the inflammatory response and poor clinical outcomes (17). Blood urea nitrogen to albumin ratio (BAR) is the ratio of blood urea nitrogen (BUN) to serum albumin. It is an easily calculable, inexpensive, and simple parameter. While BUN has long been used to evaluate kidney function, albumin is a negative acute-phase reactant. Recent studies have highlighted BAR as a predictor of mortality (18). A high BAR value has been identified as a predictor of increased mortality in patients (19). C-reactive protein (CRP) is a positive acute-phase reactant, while albumin is a negative acute-phase reactant. Serum CRP levels increase in the presence of inflammation, while serum albumin levels decrease due to malnutrition or systemic inflammation (20). Lymphocyte-to-C-reactive protein ratio (LCR), the ratio of lymphocyte count (obtained from a complete blood count) to CRP levels, is a recently studied inflammatory parameter (21).

In the present study, our objective was to determine the prevalence of hypertension in patients with GIAD and its association with aortic stiffness, including the influence of the non-dipper blood pressure pattern in patients with GIAD.

## Patients, methods and ethics committee approval

### Patient selection and methods

This prospectively designed study included 25 patients with GIAD detected in any part of the gastrointestinal system by endoscopy, colonoscopy or computed tomography, and 25 individuals without GIAD as a control group.

After obtaining consent from the patient diagnosed with GIAD, the patient's laboratory tests at the time of diagnosis were noted; the patient was taken for additional evaluation at the cardiology clinic; the patient's physical examination was performed; additional tests in the study [electrocardiography, transthoracic echocardiography, and ambulatory blood pressure monitoring (ABPM)] were applied to each patient.

During the same period, the control group also began recruiting participants; after obtaining consent from the participant, relevant laboratory tests were performed, each participant's physical examination was performed similarly to the GIAD patient group; additional tests in the study (electrocardiography, transthoracic echocardiography, and ABPM) were applied to each participant.

Inclusion criteria for the study consisted of patients aged over 18 years, with or without a diagnosis of GIAD. Exclusion criteria included the presence of malignancy, active infection, moderate to severe heart valve disease, a history of cardiac surgery, insufficient ABPMs, being a night shift worker, having sleep disorders, or being pregnant. Patients who met these exclusion criteria were not eligible for inclusion in the study. Routine laboratory tests were conducted for each patient. Hypertension was defined as the use of antihypertensive agents or an average systolic blood pressure of ≥140 mmHg or an average diastolic blood pressure of ≥90 mmHg on ABPM (22).

Demographic characteristics (age and gender), blood and serum parameters (total cholesterol (TC), low-density lipoprotein (LDL), high-density lipoprotein (HDL), triglyceride (TG), CRP, serum albumin, creatinine (Cr), alanine aminotransferase (ALT), aspartate aminotransferase (AST), hemoglobin (HG), uric acid, Na^+^, K^+^, white blood cell counts) and presence of diabetes mellitus (DM) were analyzed.

### Ethics committee approval

The study was approved by the ethics committee of the Amasya University Rectorate Non-Interventional Clinical Research Ethics Committee, Document Date and Number: 08.03.2024-184601; Board decision Number: E-76988455-050.04-184601.

### Echocardiographic evaluation

Echocardiographic evaluation was performed using a PHILIPS EPIQ 7G ultrasound system color Doppler echocardiography device. M-mode measurements and conventional Doppler measurements were utilized to determine the aortic systolic and diastolic diameters, mitral early diastolic flow velocity (E), mitral late diastolic flow velocity (A), and transmitral flow velocity ratio (E/A).

### 24 h ambulatory blood pressure measurement

All participants underwent 24 h ABPM using a MOBIL-O-GRAPH NG (Ver. 20) device. The device was set to measure blood pressure every 15 min between 07:00 a.m. and 12:00 p.m. and every 30 min between 12:00 p.m. and 07:00 a.m. At the end of the 24 h period, the device was removed, and blood pressure measurements were analyzed using a computer program. Considering the total measurements, those who successfully completed 85% or more of the measurements within the 24 h period were included in the statistical analysis.

### Statistical analysis

The statistical analysis of the study data was performed using the SPSS software (Version 22.0, SPSS, Chicago, USA). Continuous variables were expressed as mean ± standard deviation (SD), while categorical variables were presented as frequencies and percentages. The Kolmogorov–Smirnov test was used to assess the distribution pattern of the variables. For group comparisons, both the *t*-test and chi-square test were employed. *p* < 0.05 was considered statistically significant.

For the intergroup statistical analysis, categorical variables were compared using the *χ*2 test or Fisher's exact test, while continuous variables were analyzed using either the *t*-test or Mann–Whitney *U* test, based on the normality of distribution. To explore the independent association between inflammatory markers and GIAD, multivariate logistic regression analysis was employed. Odds ratios (ORs) and 95% confidence intervals (CIs) were calculated for each associated variable. Furthermore, a Receiver operating characteristic (ROC) analysis was conducted to assess the sensitivity and specificity of inflammatory markers for predicting GIAD. A *p*-value 0.05 was considered statistically significant across all analyses. Optimal cut off values for C-reactive protein to albumin ratio (CAR), CRP, BAR, NLR, LCR and SIRI were determined using ROC, with the area under the ROC curve (AUC) and 95% CI calculated for each marker (23). All statistical analyses were performed using R software version 3.6.3 (R Foundation for Statistical Computing, Vienna, Austria) MedCalc Programme and IBM SPSS Statistics version 26.0. A two-tailed *p*- value 0.05 was deemed significant in all analyses.

## Results

The mean age of the study group was 55.8 ± 16.0 years. The mean ages of the control group and patient group were similar (*p* < 0.281; Table 1). Gender distribution among the groups were similar (*p* = 0.771; Table 1). Among the participants, 11 patients (44%) had DM, while there were no differences in the number of the patients with DM between the groups (*p* > 0.05; Table 1). Moreover, GIADs were most commonly found in colon (40%) followed by duodenum (20%; Table 1).

**Table 1:** Gender distribution, mean ages and frequency of the presence of DM among the participants.

Variables	Groups/regions	*N* (%)/mean ± SD	*p*
Age	Control group	62.1 ± 9.6	0.281
	Patient group	63.8 ± 16.5	
Gender	Control group		0.771
	Male	15 (60)	
	Female	10 (40)	
	Patient group		
	Male	16 (64)	
	Female	9 (36)	
DM	Control group		0.306
	No	21 (84)	
	Yes	4 (16)	
	Patient group		
	No	18 (72)	
	Yes	7 (28)	
Region of GIAD	Duodenum	5 (20)	
	Ileum	1 (4)	
	Jejunum	1 (4)	
	Colon	10 (40)	
	Antrum (Stomach)	3 (12)	
	Cardia (Stomach)	1 (4)	
	Corpus (Stomach)	1 (4)	
	Esophagus	3 (12)	

DM, diabetes mellitus; GIAD, gastrointestinal angiodysplasia; SD, standard deviation.

TC, LDL, HDL, TG, HG, HG, WBC and CRP levels were similar in the control and patient groups (Table 2). Cr levels were significantly higher in the patient group compared to control group (*p* = 0.001; Table 2). While blood Na^+^ levels were similar between two groups, K^+^ levels were significantly higher in the patient group compared to control group (*p* = 0.021; Table 2). On the other hand, while AST levels were similar in both groups, both ALT and uric acid levels were lower in the patient group compared to control group (*p* < 0.05; Table 2).

**Table 2:** Blood and serum parameters of the patients.

Variables	Control group	Patient group	*p*
	Mean ± SD		
TC (mg/dl)	201.9 ± 53.6	180.4 ± 41.3	0.121
LDL (mg/dl)	128.2 ± 47.42	112.6 ± 31.69	0.178
HDL (mg/dl)	51.02 ± 14.72	44.92 ± 10.95	0.103
TG (mg/dl)	177.3 ± 96.71	180.3 ± 95.59	0.761
HG (g/dl)	13.48 ± 1.96	12.84 ± 1.78	0.156
WBC (×10^9^/L)	8.148 ± 2.663	7.918 ± 5.399	0.269
CRP (mg/L)	3.13 ± 2.13	12.10 ± 21.34	0.02
Cr (mg/dl)	0.7204 ± 0.1373	1.026 ± 0.5543	0.001
Na^+^ (mmol/L)	138.6 ± 1.96	140.5 ± 6.44	0.317
K^+^ (mmol/L)	4.117 ± 0.4238	4.474 ± 0.6234	0.021
AST (IU/L)	25.04 ± 15.18	25.48 ± 18.59	0.579
ALT (U/L)	27.52 ± 14.91	21.10 ± 24.32	0.006
Uric acid (mg/dl)	7.751 ± 10.34	5.128 ± 2.031	0.047
NLR	1.54 ± 0.68	3.82 ± 5.94	0.011
BAR	0.53 ± 0.108	1.22 ± 0.83	<0.0001
SIRI	0.97 ± 0.73	1.86 ± 2.14	0.021
SII	438 ± 157.0	842 ± 246.80	0.146
CAR	0.07 ± 0.05	0.40 ± 0.93	0.014
LCR	1.82 ± 3.03	0.59 ± 0.62	0.020
MLR	0.284 ± 0.239	0.346 ± 0.180	0.006

ALT, alanine aminotransferase; AST, aspartate aminotransferase; BAR, blood urea nitrogen to albumin ratio; CAR, C-reactive protein to albumin ratio; Cr, creatinine; CRP, C-reactive protein; HDL, high-density lipoprotein; HG, hemoglobin; LDL, low-density lipoprotein; LCR, lymphocyte-to-C-reactive protein ratio; MLR, monocyte-to-lymphocyte ratio; NLR, neutrophil-to-lymphocyte ratio; SD, standard deviation; SII, systemic immune-inflammation index; SIRI, systemic immune response index; TC, total cholesterol; TG, triglycerides; WBC, white blood cell count.

Systolic blood pressure (SBP) and diastolic blood pressure (DBP) measurements in the morning and at night did not differ between the groups (Table 3). Similarly, ejection fraction (EF) and mitral valve doppler E wave measurements did not differ between the groups (Table 3). However, mitral valve doppler A measurements were found significantly higher, while E/A measurements were significantly lower, in the patient group compared to control group (*p* < 0.0001; Table 3). Aortic systolic and aortic diastolic measurements were significantly higher in the patient group compared to control group (*p* = 0.006; Table 3). While aortic strain and aortic distensibility were lower, elastic module and degree of aortic stiffness were significantly higher in the patient group compared to control group (*p* < 0.0001; Table 3). Left ventricular thickness was significantly higher in the patient group compared to control group (*p* < 0.0001; Table 3). Left ventricular hypertrophy (LVH), as well as non-dipper blood pressure pattern, was significantly more frequent in the patient group (*p* < 0.0001; Table 3).

**Table 3:** Echocardiography and ABPM measurements of the patients.

Variables	Control group	Patient group	*p*
	Mean ± SD/*N* (%)		
SBP morning (bpm)	121.56 ± 13.40	116.56 ± 11.97	0.170
SBP night (bpm)	105.7 ± 11.19	113.04 ± 18.10	0.159
DBP morning (bpm)	76.96 ± 12.22	71.56 ± 9.31	0.085
DBP night (bpm)	71.76 ± 11.51	67.04 ± 11.8	0.160
EF (%)	61.20 ± 2.18	60.28 ± 3.69	0.536
Mitral valve doppler E (m/sec)	0.736 ± 0.140	0.740 ± 0.16	0.536
Mitral valve doppler A (m/sec)	0.652 ± 0.120	0.864 ± 0.170	<0.0001
E/A	1.136 ± 0.200	0.871 ± 0.221	<0.0001
Aortic systolic diameter (cm)	3.218 ± 0.080	3.496 ± 0.483	0.006
Aortic diastolic diameter (cm)	2.834 ± 0.083	3.303 ± 0.494	0.006
Aortic strain (%)	13.504 ± 0.760	5.779 ± 2.486	<0.0001
Elastic module (mmHg)	3.266 ± 0.660	10.279 ± 7.539	<0.0001
Degree of aortic stiffness (m/sec)	0.117 ± 0.009	0.370 ± 0.267	<0.0001
Aortic distensibility (cm^2^.dyn^−1^.10^−6^)	0.629 ± 0.11	0.257 ± 0.128	<0.0001
Left ventricular wall thickness (cm)	0.984 ± 0.117	1.292 ± 0.050	<0.0001
LVH			
Yes	2 (8)	25 (100)	<0.0001
No	23 (92)	0 (0)	
Dipper	24 (96)	4 (16)	<0.0001
Non-dipper	1 (4)	21 (84)	

ABPM, ambulatory blood pressure monitoring; DBP, diastolic blood pressure; E/A, transmitral flow velocity ratio; EF, ejection fraction (%); LVH, left ventricular hypertrophy; Mitral A, late diastolic flow velocity; Mitral E, early diastolic flow velocity; SBP, systolic blood pressure; SD, standard deviation.

**Figure 1:**
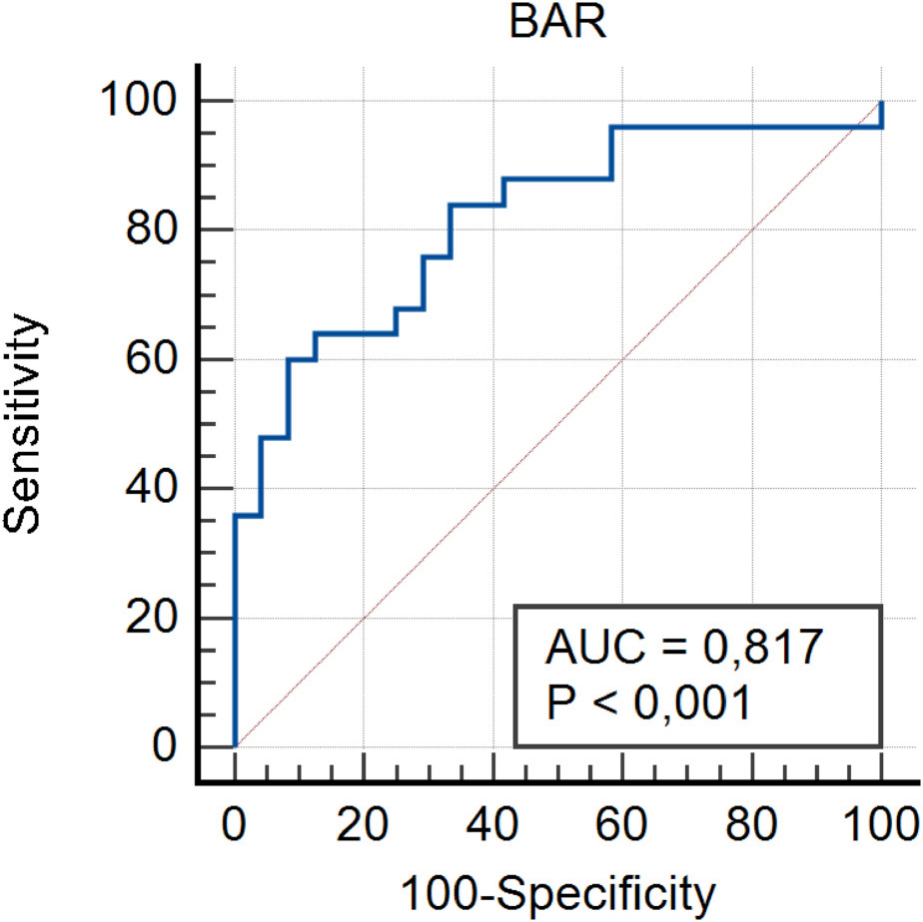
Receiver operating curve for determining the optimal cut-off values for BAR. BAR, blood urea nitrogen to albumin ratio.

**Figure 2:**
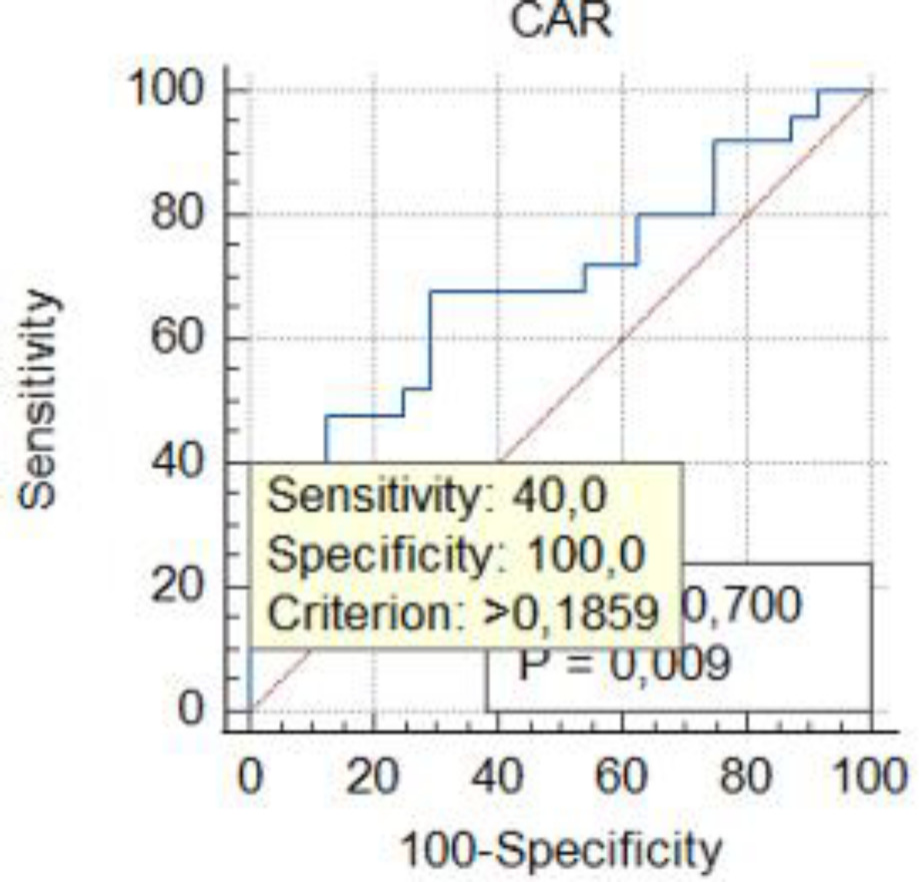
Receiver operating curve for determining the optimal cut-off values for CAR. CAR, C-reactive protein to albumin ratio.

**Figure 3:**
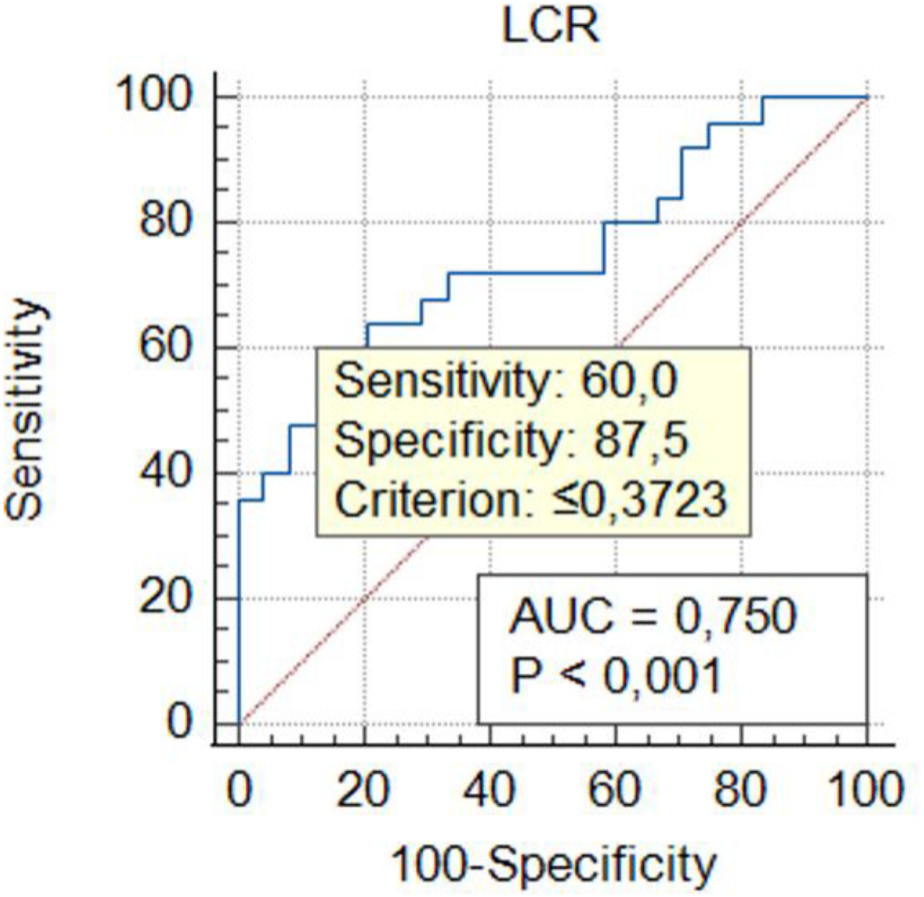
Receiver operating curve for determining the optimal cut-off values for LCR. LCR, lymphocyte-to-C-reactive protein ratio.

**Figure 4:**
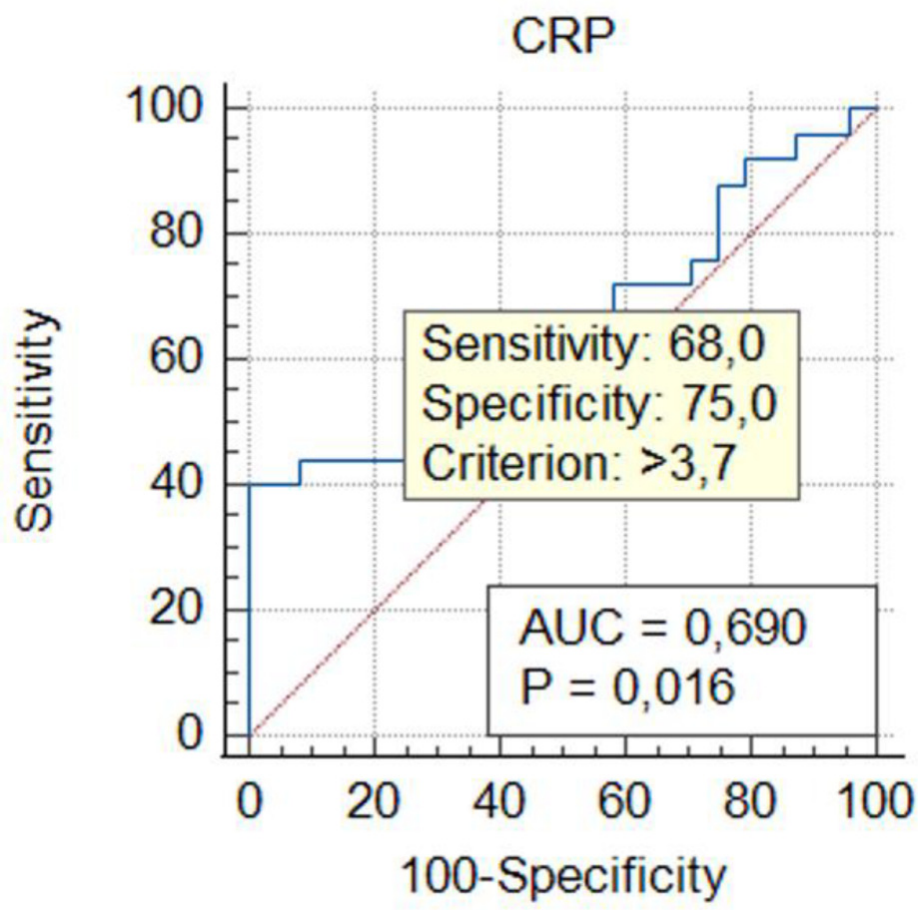
Receiver operating curve for determining the optimal cut-off values for CRP. CRP, C-reactive protein.

**Figure 5:**
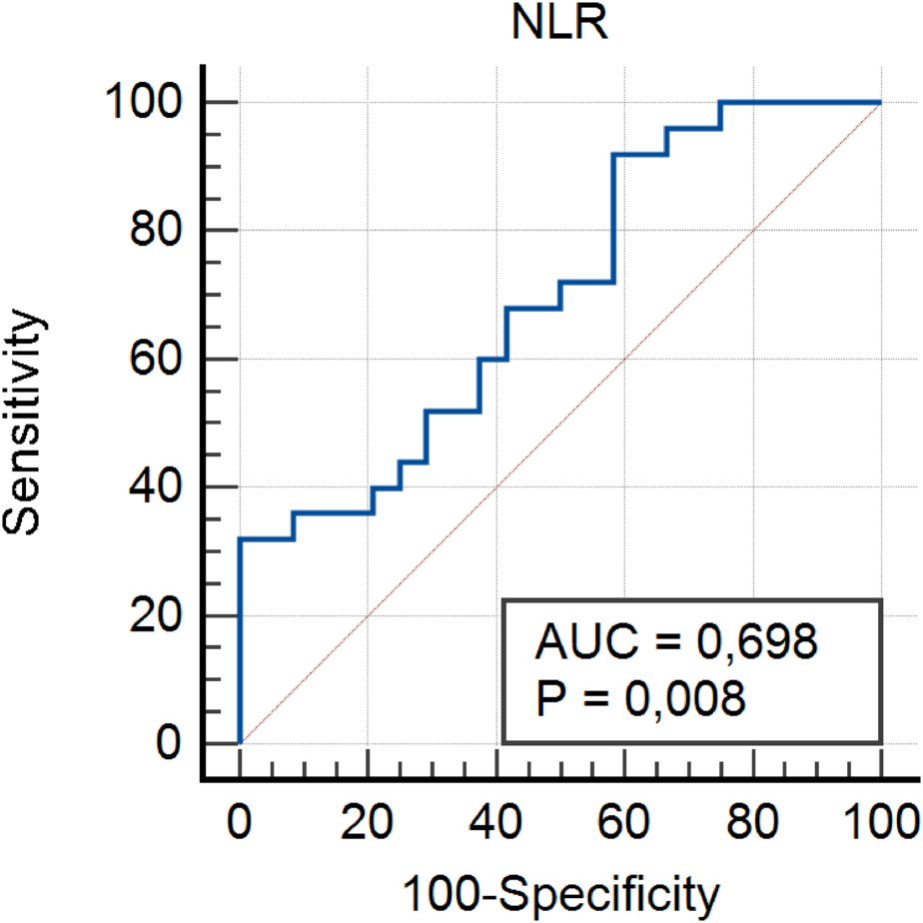
Receiver operating curve for determining the optimal cut-off values for NLR. NLR, neutrophil-to-lymphocyte ratio.

Threshold values for inflammatory markers in GIAD patients were determined using ROC analysis to assess their predictive properties. By calculating the sensitivity and specificity of the model with ROC analysis, the AUC was obtained, which indicates the model's accuracy in correctly classifying GIAD based on laboratory data. The AUC values and the optimal threshold values are presented in Figures 1–5 and Table 4, respectively. The AUC for BAR was found to be 0.81 (95% CI, 0.68–0.91), demonstrating the high accuracy of BAR in predicting the diagnosis of GIAD. The AUC for LCR was calculated as 0.75 (95% CI, 0.60–0.86), while the AUC for SII was 0.70 (95% CI, 0.55–0.82). For CAR, the AUC was determined to be 0.70 (95% CI, 0.55–0.83), indicating its predictive power in the diagnosis of GIAD. The AUC for CRP was 0.69 (95% CI, 0.54–0.81), suggesting that this parameter is also a predictor for GIAD. The AUC for NLR was calculated as 0.69 (95% CI, 0.55–0.82), and the AUC for SIRI was 0.53 (95% CI, 0.39–0.68).

**Table 4:** The summary of predictive ability of biochemical markers and ratios.

Variables	AUC	95% CI	Sensitivity (%)	Specifcity (%)	Youden index	Cut-off value	*p*-value
BAR	0.81	0.68–0.91	60.00	91.67	0.51	0.021	**<0** **.** **0001**
CRP	0.69	0.54–0.81	68.00	75.00	0.43	3.7	**0**.**015**
CAR	0.70	0.55–0.83	40.00	100	0.40	0.185	**0**.**008**
LCR	0.75	0.60–0.86	60.00	87.50	0.47	0.372	**0**.**0004**
NLR	0.69	0.55–0.82	32	100	0.33	2.72	**0**.**008**
SII	0.70	0.55–0.82	40	95	0.35	2.91	**0**.**0064**
SIRI	0.53	0.39–0.68	48	66	0.14	3.73	0.65

BAR, blood urea nitrogen to albumin ratio; CAR, C-reactive protein to albumin ratio; CRP, C-reactive protein; LCR, lymphocyte-to-C-reactive protein ratio; NLR, neutrophil-to-lymphocyte ratio; SII, systemic immune-inflammation index; SIRI, systemic immune response index.

*p*-values shown in bold indicate statistically significant results.

To further investigate the relationship between GIAD and inflammatory markers, univariate and multivariate regression analyses were performed. In the univariate logistic regression analysis, the risk of GIAD was evaluated for each inflammatory marker, and variables found to be significant were included in the multivariate logistic regression analysis (Table 5). The results showed statistically significant associations between GIAD and BAR ≥ 0.021 (*p* = 0.0001; OR: 6.01; 95% CI: 1.25–10.45), CAR ≥ 0.185 (*p* = 0.021; OR: 4.03; 95% CI: 2.74–18.33), LCR ≥ 0.372 (*p* = 0.018; OR: 1.01; 95% CI: 1.03–1.56), CRP > 3.7 (*p* = 0.021; OR: 1.27; 95% CI: 1.08–1.56), and NLR ≥ 2.72 (*p* = 0.029; OR: 2.40; 95% CI: 1.08–5.35). These analyses suggest that when BAR levels exceed 0.021, each unit increase predicts approximately a six-fold increase in the risk of developing GIAD, while CAR levels exceeding 0.185 predict a four-fold increase. Similarly, LCR levels above 0.372, CRP levels above 3.7, and NLR levels above 2.72 predict approximately one-fold, 1.2-fold, and 2.4-fold increases in risk, respectively. These findings highlight the predictive ability of CAR for GIAD and suggest its potential as a biomarker in clinical applications. Furthermore, they underscore the significant roles of CAR and BAR in the disease's pathophysiology, indicating that evaluating these markers together could enhance diagnostic accuracy. In conclusion, inflammatory markers such as CAR and BAR could be utilized in clinical practice to assess GIAD risk and enable early diagnosis. With their high sensitivity and specificity, these markers hold promise as valuable biomarkers in the diagnosis and prognosis of GIAD.

**Table 5:** Multivariate logistic regression model for the association between GIAD and inflammatory markers.

Variable	Odds Ratio	(95% CI)	*p*-value
BAR	6.01	1.25–10.45	0.0001
CAR	4.03	2.74–18.33	0.021
LCR	1.01	0.138–0.83	0.018
CRP	1.27	1.038–1.56	0.021
NLR	2.40	1.081–5.35	0.009

BAR, blood urea nitrogen to albumin ratio; CAR, C-reactive protein to albumin ratio; CRP, C-reactive protein; GIAD, gastrointestinal angiodysplasia; LCR, lymphocyte-to-C-reactive protein ratio; NLR, neutrophil-to-lymphocyte ratio.

## Discussion

Despite some important limitations discussed below, this study underscores the complex interplay between GIAD and cardiovascular comorbidities, revealing critical insights into their shared pathophysiology and clinical implications.

We observed that GIADs are predominantly localized within the colon, with significant associations observed between GIAD and a spectrum of cardiovascular comorbidities, including hypertension, increased aortic stiffness, and LVH. Moreover, the prevalence of non-dipper hypertension was notably higher in patients with GIAD, contributing to increased cardiovascular risk. Our findings are similar to previous studies reporting that GIAD is rarely detected under the age of 50, is usually seen in patients over 60, and approximately two-thirds of cases are seen in patients aged 70 and older. This provides insight into why comorbidities are higher in the GIAD group (6). Elevated serum creatinine and potassium levels, indicative of renal impairment, were also observed, underscoring the systemic nature of the disease.

GIAD is the major vascular malformation, especially within the cecum and ascending colon, and can lead to significant GI bleeding, particularly in older adults and those with underlying cardiovascular conditions (24). In our study, we found that GIADs were primarily localized in the colon (40%), followed by the duodenum (20%). These findings are consistent with previous literature reporting that the colon, especially the cecum and ascending colon, as regions frequently involved due to decreased blood flow velocity and elevated vascular susceptibility (1). The anatomical propensity of these areas to develop angiodysplastic changes can be attributed to factors including regional hypoperfusion and hypoxia, both of which promote endothelial proliferation and capillary dilation (25, 26). Besides the anatomical and physiological factors, the age-related degeneration of small blood vessels is also a recognized factor for the development of GIAD, particularly in older populations (27). The cumulative effects of hypoperfusion and hypoxia over time may lead to degenerative changes in the vascular architecture, facilitating the emergence of angiodysplastic lesions in these regions of the colon.

In a retrospective cohort study conducted in Portugal in 2023, it was found that the presence of small bowel angioectasia alone did not affect survival, but comorbidities and age were independent predictors of poor survival (28). Our study demonstrated that patients with GIAD had a significantly increased prevalence of cardiovascular comorbidities, including hypertension, aortic stiffness, and LVH. This observation is corroborated by multiple studies in the literature, which underscore a significant association between gastrointestinal vascular malformations and systemic cardiovascular disorders (29, 30). The coexistence of these conditions can be attributed to overlapping pathophysiological mechanisms, such as chronic hypertension and arterial stiffness. In our cohort, GIAD patients exhibited significantly increased aortic systolic and diastolic diameters compared to controls, indicating greater vascular rigidity. These findings are consistent with those reported previously as hypertension can exacerbate the development of GIAD by increasing mechanical stress on already vulnerable vascular structures (31, 32). Moreover, it can be proposed that aortic stiffness, compounded by hypertension, leads to heightened pulse wave velocity and arterial impedance, ultimately increasing the risk of vascular malformations (33, 34). This indicates that cardiovascular comorbidities complicate the management of GIAD, highlighting the need for a thorough cardiovascular assessment in these patients.

Non-dipper pattern has been strongly correlated with increased cardiovascular complications, including LVH and enhanced aortic stiffness (35, 36). In our study, we observed a high prevalence of the non-dipper blood pressure pattern in patients with GIAD. This was previously described as indicative of autonomic dysfunction, involving reduced parasympathetic tone and heightened sympathetic activity, which contributes to sustained nocturnal hypertension (13). Our findings align with these associations, as patients with a non-dipper profile exhibited more frequent LVH, reduced aortic compliance, and greater vascular stiffness. Thus, our findings suggests that non-dipper hypertension not only serves as an important marker of cardiovascular risk but also actively contributes to the pathogenesis of GIAD through sustained endothelial stress. Incorporating ABPM into routine practice for GIAD patients may be important for the early identification of non-dipper patterns and for optimizing antihypertensive therapy to mitigate cardiovascular risk. LVH is typically a response to chronic hypertension and increased systemic vascular resistance (37), both of which were more prevalent in the patient group in our study. The reduction in the E/A ratio, as well as an increased A wave velocity, suggests impaired diastolic function, which may predispose these patients to heart failure with preserved ejection fraction (38, 39). Previous studies revealed a correlation between LVH, diastolic dysfunction, and increased cardiovascular morbidity and mortality (40, 41). The impaired left ventricular compliance and function observed in our study indicates the importance of echocardiographic screening for early detection of LVH in GIAD patients, particularly those with coexisting hypertension, to prevent adverse cardiovascular outcomes.

Biochemical analysis revealed that patients with GIAD had significantly elevated serum creatinine and potassium levels compared to controls, suggesting impaired renal function (42). It was previously found that renal dysfunction is common among patients with cardiovascular comorbidities, including GIAD (43, 44). The elevated serum creatinine levels may reflect decreased renal perfusion as a consequence of systemic vascular rigidity, thereby contributing to the overall cardiovascular burden in these patients (45, 46). Interestingly, reduced ALT and uric acid levels were observed in the patient group, which may indicate underlying hepatic dysfunction and altered oxidative metabolism. Although lower uric acid levels are not typical in cardiovascular disease, it is possible that chronic hypoperfusion and increased oxidative stress result in altered uric acid dynamics (47). Elevated uric acid levels are often associated with metabolic syndrome and kidney dysfunction, however, in the context of GIAD, a reduction in uric acid levels may suggest altered oxidative metabolism or renal clearance disturbances (48). On the other hand, the reduction in ALT may reflect diminished hepatic function or hepatocyte injury, which is often exacerbated by the hemodynamic changes associated with aortic stenosis (49). Additional studies investigating the role of renal and hepatic dysfunction in the pathophysiology of GIAD, and whether these biochemical parameters could serve as prognostic indicators for disease progression.

Recent evidence suggests a link between gut microbiota dysbiosis, vascular aging, and cardiovascular disease. This phenomenon has been termed the “gut-heart axis.” The transfer of bacterial products into the bloodstream has been linked to increased arterial stiffness, atherosclerosis, hypertension, and cardiovascular risk in humans. The absolute impact of inflammation at every stage of the process is undeniably evident (50). In this study, the relationship between GIAD and inflammatory markers was thoroughly evaluated, and the obtained results were compared with the existing literature. The findings indicate that inflammatory markers such as CAR, BAR, LCR, CRP, and NLR play a significant role in predicting the risk of GIAD. However, we found that SIRI, a strong predictor of vascular disease, had relatively low sensitivity in our study. This finding suggests that inflammatory processes are actually more complex than previously thought. Specifically, increases in CAR and BAR levels were found to significantly raise the risk of developing GIAD, suggesting that these two markers may have critical roles in the pathophysiology of the disease.

Although previous studies have highlighted the impact of inflammation on the pathophysiology of GIAD, this study is the first to comprehensively address the potential usability of specific inflammatory markers as biomarkers in this context. For instance, CAR has been identified as a strong predictor of GIAD due to its high sensitivity and specificity. Similarly, BAR emerges as a valuable tool for better understanding the impact of inflammation on the disease and predicting its progression (16). In this study, SIRI values analyzed through ROC analysis (AUC: 0.722, 95% CI: 0.665–0.779) were found to be associated with poor clinical outcomes. The cut-off value for SIRI was determined as 2.676, with a sensitivity of 0.595, specificity of 0.751, PPV (positive predictive value) of 0.217, and NPV (negative predictive value) of 0.394. Similarly, in our study, we identified a comparable cut-off value of 3.73 (AUC: 0.53, 95% CI: 0.39–0.68, sensitivity = 0.48, specificity = 0.66).

The significant differences in other elements of the inflammatory response between the GIAD group and the control group appear to be consistent with this finding. In the study of Mirna et al. (51), the median NLR was reported as 2.48 (IQR 1.55–4.58), with an optimal cut-off value for NLR calculated using the Youden Index as 4.00 (sensitivity: 63%; specificity: 86%; PPV: 71%; NPV: 80%). In our study, the median NLR value in the GIAD patient group was 3.82 ± 5.94, and we determined a cut-off value of 2.72 for NLR (AUC: 0.69; 95% CI: 0.55–0.82; sensitivity: 0.32; specificity: 1; Youden Index: 0.33). Consistent with previous findings, we also observed that the NLR ratio in the patient group was significantly higher than that in the control group. In our study, the NLR ratio in the patient group was also found to be significantly higher than that in the control group (52). In our study, the BAR value was found to have an AUC (95% CI) of 0.81 (0.68–0.91) with a cut-off value of 0.021. Previous studies reported BAR values with an AUC of 0.710 (95% CI: 0.668–0.751), a cut-off of 7.43, and a *p*-value < 0.001, with a sensitivity of 67%. Similarly, the elevated CAR value indicates the severity of the inflammatory event. In a study, a CAR value ≥ 0.18 was associated with the worst prognosis in patients undergoing chemotherapy (HR = 3.132; *p* < 0.001) (53). In our study, the CAR value in the GIAD group was also significantly higher than in the control group, with a cut-off value of 0.185. LCR, as highlighted in other studies, is considered a protective parameter against adverse inflammatory responses (21). Low LCR values have been shown to indicate an increased risk in vascular inflammatory diseases, including coronary artery disease (CAD) (54). In a study conducted by Nakamura et al. (20), the best cut-off value for CRP in their inflammation group was identified as 2.0 mg/dl. Similarly, in our study, the CRP level in the GIAD patient group was significantly higher, with a cut-off value of 3.7, consistent with the findings of previous studies.

These results suggest that the use of markers such as CAR and BAR in clinical practice could contribute to the early diagnosis and improved prognosis of GIAD. Particularly, the non-invasive nature of these markers enhances their applicability across large patient populations and provides clinicians with valuable guidance for individualized patient management.

In conclusion, this study demonstrates that inflammatory markers such as CAR and BAR can be utilized in the diagnosis and prognosis of GIAD in clinical applications. These findings may shed light on the management and treatment planning of GIAD. In conclusion, this study highlights the significant association between GIAD and cardiovascular comorbidities, including hypertension, increased aortic stiffness, and LVH. The prevalence of the non-dipper blood pressure pattern in GIAD patients further complicates their cardiovascular risk profile, underscoring the need for comprehensive cardiovascular evaluation and targeted antihypertensive management. Routine echocardiographic assessment and ABPM may serve as valuable tools in detecting early cardiovascular changes and optimizing patient outcomes.

This study highlights the intricate relationship between GIAD and cardiovascular comorbidities. The identification of non-dipper hypertension and systemic inflammatory markers as critical contributors offers new avenues for diagnostic and therapeutic interventions. Routine cardiovascular and biochemical assessments, combined with innovative biomarkers like CAR and BAR, can significantly enhance patient outcomes. Future research should aim to refine these findings and explore their applications in broader clinical contexts.

## Limitations and future directions

While this study provides valuable insights, it has significant limitations. The single-center design, limited sample size, and focus on only specific markers may limit the generalizability of the results. Therefore, larger, multi-center, prospective studies with more participants from diverse groups are needed to confirm these findings and increase their generalizability. Expanding the scope to include additional biomarkers and longitudinal follow-up could further elucidate the pathophysiology of GIAD and its systemic associations.

## Data Availability

The raw data supporting the conclusions of this article will be made available by the authors, without undue reservation.
